# A Rare Finding of a BRAF Mutation in Renal Cell Carcinoma with Response to BRAF-Directed Targeted Therapy

**DOI:** 10.7759/cureus.449

**Published:** 2016-01-06

**Authors:** Natasha Banerjee, Esha Sachdev, Robert A Figlin

**Affiliations:** 1 Oncology, Cedars-Sinai Medical Center; 2 Internal Medicine, Cedars-Sinai Medical Center; 3 Oncology, Cedars-Sinai Medical Center, Samuel Oschin Comprehensive Cancer Institute

**Keywords:** oncology, targeted therapy, renal cell cancer, whole genome sequencing, braf inhibitor, precision medicine

## Abstract

Whole exome sequencing can identify somatic mutations in malignant tumors and allow for personalized and novel treatment of common malignancies. Mutations in the BRAF gene are rare in renal cell carcinoma, and thus, BRAF inhibitors are not considered standard in the treatment of these cancers. Here, we report a case of a patient with a rare BRAF-mutated metastatic renal cell carcinoma who obtained a good clinical response to BRAF inhibition. This case underscores the value of precision medicine in an era of rapidly evolving therapeutics for malignancies.

## Introduction

The field of oncology is rapidly changing with the introduction of targeted therapies for tumor-specific genetic mutations. Whole exome genetic sequencing has made it possible to rapidly identify targetable mutations and allow for novel treatments of rare or aggressive malignancies. Here, we report a case of a patient with BRAF-mutated metastatic renal cell carcinoma who obtained a good clinical response to BRAF inhibition.

## Case presentation

A 63-year-old male initially presented with a six-month history of left-sided scrotal edema and increased urinary frequency. A renal ultrasound was performed and demonstrated a 6 cm mass adjacent to the left kidney. Subsequent computed tomography of the abdomen and pelvis showed a 4.7 cm enhancing mass involving the mid to upper pole of the left kidney with bulky left periaortic lymphadenopathy measuring up to 7.2 centimeters. Computed tomography of the chest was done and showed a 1.5 cm suprahilar mass, a 2.4 cm left infrahilar mass, and multiple left-sided subcentimeter pulmonary nodules. Magnetic resonance imaging of the brain revealed no evidence of metastatic disease. Informed patient consent was obtained for treatment.

A core needle biopsy of the left retroperitoneal lymph node mass was done and demonstrated an undifferentiated carcinoma with pleomorphic features. The immunohistochemical profile was compatible with renal cell carcinoma and initial analysis suggested sarcomatoid features. The patient was initiated on treatment for metastatic renal cell carcinoma with sunitinib and gemcitabine and completed two cycles of therapy with stable disease as the best response. Next generation sequencing was performed by the Cedars-Sinai pathology department with a panel designed to identify hotspot mutations in 50 genes frequently mutated in cancers. This analysis of the patient’s tumor revealed an activating V600E BRAF mutation. No other significant mutations were found in the panel, which included analysis of the ATM, KIT, KRAS, MET, PIK3CA, PTEN, TP53, SRC, and VHL genes. As a result, sunitinib and gemcitabine were stopped, and the patient was initiated on therapy with vemurafenib, a BRAF V600 inhibitor.

Follow-up imaging with computed tomography was performed three months after starting vemurafenib. The target lesions showed a response to therapy with a decrease in the size of the renal mass from 4.7 to 4.1 cm and a decrease in the left retroperitoneal conglomerate lymphadenopathy from 9.6 to 5.8 centimeters. The pulmonary nodule at the left lower lobe decreased from 20 to 12 mm with the remaining nodules being stable in size. While on treatment, the patient experienced fatigue, mild dyspnea on exertion, and a squamous cell carcinoma of the scalp, consistent with known side-effects of the BRAF inhibitors.

Due to good clinical response to therapy, the patient underwent cytoreductive left radical nephrectomy with retroperitoneal lymph node dissection. Pathology confirmed renal cell carcinoma with areas of papillary architecture as well as sarcomatoid and undifferentiated components in both the kidney and lymph nodes. Next generation sequencing was repeated on the tumor sample and again returned positive for an activating mutation in the BRAF V600E gene. The patient was continued on BRAF inhibition postoperatively and continued to derive clinical benefit.

## Discussion

The BRAF gene is located on chromosome 7 and encodes BRAF, a signaling protein downstream of Ras that activates the MAP-kinase pathway and is implicated in cell proliferation and differentiation. Activating mutations in the BRAF proto-oncogene results in constitutive activation of the mitogen-activated protein kinase (MAPK) pathway and subsequent tumorigenesis. The V600E mutation causes an amino acid substitution at position 600 in exon 15 of the BRAF gene, resulting in the replacement of valine (V) by glutamic acid (E).

Our case is unique because, in the literature, mutations in the BRAF oncogene are not implicated in the development of renal cell carcinoma and are extremely rare. The NCI’s cancer genome atlas (TCGA) performed a molecular characterization of clear cell renal carcinoma using whole exome sequencing of over 400 tumor samples. Analysis of the mutation data identified 19 significantly mutated genes, with VHL, PBRM1, SETD2, PTEN, MTOR, and TP53 being among the most common. Mutations in BRAF were not identified as significant [1]. In addition, a study analyzing allelic changes at the BRAF locus in 50 renal cell cancer patients (20 papillary, 15 clear cell, and 15 chromophobe RCCs) found no mutations at exon 11 or 15 of BRAF in any of the patients [2]. Similarly, in another study involving 121 renal cell cancer patients, none of the clear cell RCC cases had mutations in BRAF [3].

While it is well-established that mutations in the von Hippel-Lindau (VHL) gene and mammalian target of rapamycin (MTOR) pathway are involved in the tumorigenesis of renal cell carcinomas, the role of BRAF in these cancers has not been elaborated [4]. Therefore, treatment with a BRAF inhibitor would not be expected to yield a positive response in an unselected patient. In the case of the patient described in this report, the standard treatment of metastatic renal cell carcinoma with sunitinib and chemotherapy yielded only stable disease, while targeted therapy against the BRAF mutation with vemurafenib resulted in a partial response. The patient then went on to receive cytoreductive surgery, allowing for an improved outcome and likely improved overall survival. 

## Conclusions

The use of next generation sequencing and identification of a BRAF mutation in this patient allowed for personalized and targeted treatment against a mutation that had previously not been implicated in renal cell carcinoma. To our knowledge, this is the first reported case of a renal cell carcinoma responding to BRAF inhibitor therapy. Our case underscores the value of precision medicine in a rapidly evolving era of employing targeted therapies against malignancies.  
